# Interstitial Cystitis or Painful Bladder Syndrome in a Premenopausal Female Precipitated by Oral Combined Contraceptives

**DOI:** 10.7759/cureus.8348

**Published:** 2020-05-29

**Authors:** Anna Thompson, Ashley E Siegel, Zachery Thompson, John M Tramont

**Affiliations:** 1 Pediatrics, University of Central Florida College of Medicine, Orlando, USA; 2 Medicine, University of Central Florida College of Medicine, Orlando, USA; 3 Clinical Sciences, University of Central Florida College of Medicine, Orlando, USA

**Keywords:** interstitial cystitis, painful bladder syndrome, ocps

## Abstract

It has been well documented that female sex is a significant risk factor for the development of various autoimmune diseases. While the reason for this has been debated, one well-regarded theory is that increased estrogen and decreased testosterone play a role in this predisposition. Interstitial cystitis (IC), also known as painful bladder syndrome (PBS), is an autoimmune disorder that affects over nine million women in the United States. It presents with pelvic and bladder pain and urinary symptoms, both of which significantly and negatively affect the quality of life. Even so, very few studies have examined the pathophysiologic relationship between autoimmune disorders and hormonal contraceptives. In this report, we present a case of IC likely precipitated by oral contraceptives (OCPs) in a premenopausal female. Shortly after beginning OCPs, this patient developed symptoms of severe pelvic pain and increased urinary frequency. Over the course of a year, the patient was diagnosed and treated for a variety of conditions, such as urinary tract infection (UTI), fungal vaginitis, and nephrolithiasis. After consultation with a gynecologist, a normal abdominal CT scan, and unsuccessful cystoscopy due to pain, she was finally diagnosed with IC. The patient independently learned of a potential link between hormonal contraceptive pills and IC and decided to discontinue this method of birth control. Following this, her symptoms completely resolved within several months. The timing of her initiation and discontinuation of OCPs, alongside her symptomatology, suggest a connection to the development of IC. A literature review was performed, which supports this association. We, therefore, highlight this case as an important example of IC precipitated by OCPs.

## Introduction

Interstitial cystitis (IC), also referred to as painful bladder syndrome (PBS), is a chronic pain condition that affects more than nine million women in the United States [1,2]. The International Society for the Study of Bladder Pain Syndrome defines this condition as “chronic pelvic pain, pressure, or discomfort perceived to be related to the urinary bladder, accompanied by at least one other urinary symptom such as a persistent urge to void or urinary frequency”; a myriad combination of symptoms can make this disease difficult to diagnose [3]. Though there is no clear consensus, a number of etiological theories have been proposed for IC, which include alterations in the urothelium and epithelial barrier functions that allow the permeation of toxic metabolites, alteration of pain perception pathways, and innate predisposition to autoimmune diseases, such as through positive family history [3-5].

It is well established that autoimmune diseases are more prevalent in women than men, with some diseases affecting women at a 7:1 ratio over men [6]. This skew is likely due to the effect of estrogens and progestins on the immune system. A study from 1949 by Hand et al. examined 204 women with IC and found that 36 of them experienced fluctuations of PBS in time with the menstrual cycle [7]. While the study is dated, more recent sources similarly document a premenstrual flare of pain associated with IC [1,8]. This suggests that estrogen and progesterone (the major fluctuating hormones during the menstrual cycle) may play a role in the etiology and pathophysiology of IC/PBS, likely through their roles in testosterone reduction and prostaglandin production, respectively. Similarly, in animal models, it has been found that estrogens stimulate Th2 cell predominance and secretion of type 2 cytokines, while androgens promote Th1 cell predominance [6,9]. Robust Th2 cell responses have been linked to various autoimmune conditions, such as lupus, autoimmune glomerulonephritis, multiple sclerosis, and possibly IC [10,11]. In addition, it has been reported that there may be a possible link between autoimmune disease and oral contraceptives (OCPs) [12]. It is not a stretch, therefore, to reason that the estrogen and progesterone in OCPs may play a role in the development of IC/PBS in susceptible women, likely by lowering testosterone levels. 

In this case report, we present a case of IC that developed in a premenopausal female shortly after beginning OCPs; we also engage in a literature review focusing on existing evidence supporting the link between hormonal contraception and IC. The authors first encountered this patient in an outpatient obstetrics and gynecology clinic and obtained her preceding history through interviews and clinical encounter notes.

## Case presentation

An 18-year-old, previously healthy female presented to the emergency department (ED) with sudden-onset sharp, burning pain described as “glass shards in her abdomen” for two weeks. The pain was localized to the lower abdomen and groin, was exacerbated by urination and movement, and was alleviated only by high water intake. The patient also reported bright red blood in her urine, urinary frequency, and dysuria during this time. The patient had no prior history of similar symptoms. Family history was negative for any renal or bladder conditions or autoimmune disease. The patient was not sexually active and had never been sexually active in the past. She denied any symptoms of vaginal pain. Her only medication was a combined oral contraceptive pill (TriNessa: norgestimate and ethinyl estradiol), which she had started taking two weeks prior to symptom onset. Evaluation by ED staff included appropriate laboratory studies and a physical examination, including pelvic examination. Urinalysis showed microscopic hematuria, but other routine laboratory results were within normal ranges. She was discharged from the ED with a diagnosis of acute urinary tract infection (UTI) and a 10-day course of ciprofloxacin, with instructions to follow-up with her primary care clinician. Per patient, her symptoms did not improve following treatment with antibiotics. Since discharge, she reported urinating 60+ times per day with pelvic pain and an incomplete voiding sensation. Her intermittent pelvic pain progressively developed into constant pain over the next few months.

She subsequently visited various physicians within the specialties of family and internal medicine and urology for second opinions regarding the etiology of her symptoms, many who had different diagnoses and treatment plans. These included: UTI managed with nitrofurantoin, nephrolithiasis managed with phenazopyridine for pain management, and fungal vaginitis managed with nystatin cream. She experienced no symptomatic improvement with any of these treatments.

**Figure 1:**
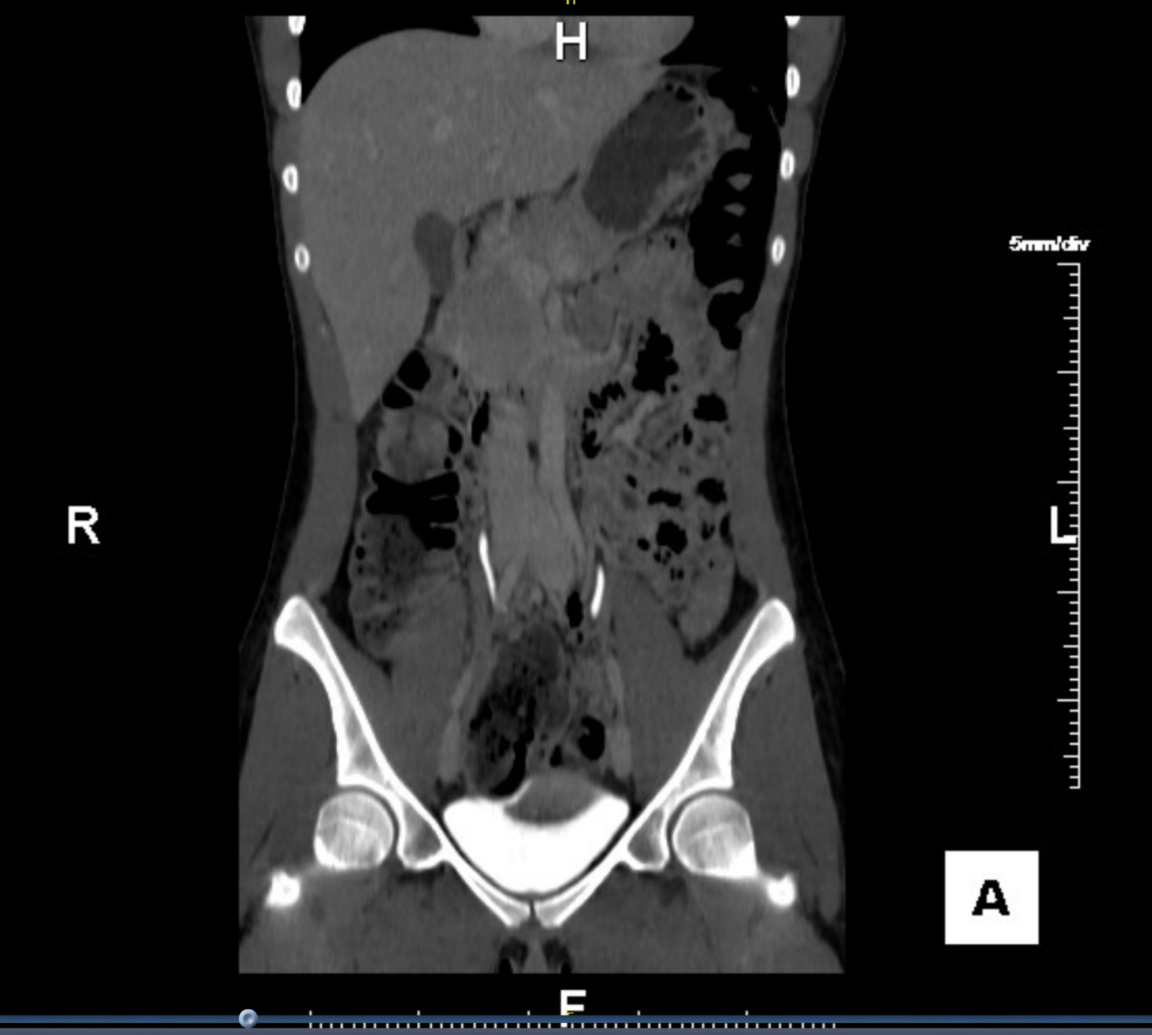
Normal abdominal and pelvic CT scan - coronal view CT: computed tomography

Approximately one year after the symptom onset, she was referred to our obstetrics and gynecology clinic. An abdominal and pelvic CT scan was ordered but showed no abnormalities (Figure 1). Outpatient cystoscopy was attempted but immediately terminated due to extreme pain. Even without cystoscopy imaging, the patient’s normal anatomy along with her symptomatology led to a diagnosis of chronic IC. She was advised to avoid triggers, encouraged to continue high fluid intake, and prescribed a higher dose of phenazopyridine for bladder relaxation and pain relief.

Through independent online searching and article browsing, the patient learned of a possible link between IC and OCPs and decided to discontinue her OCP. Following this change, the patient reported a drastic improvement in symptoms. Within four months of OCP discontinuation, she experienced an almost-complete resolution of her consistent pain, with only two subsequent flare-ups of pelvic pain and urinary frequency. Within seven months, she reported full resolution of all symptoms.

## Discussion

This case demonstrates an association between OCPs and IC, as illustrated by the timing of the patient’s OCP use and the development of her pain and urinary symptoms. Additionally, the resolution of her symptoms that followed the discontinuation of OCPs further supports this association. However, it is important to note that this association is based on subjective information provided by the patient. In addition, her symptom resolution coincided not only with the cessation of her OCPs but also with appropriate diagnosis and pain treatment. Hence, it is possible that the relationship between OCPs and IC may represent correlation rather than causation. Regardless of this, patients with IC who are treated for pain traditionally continue to experience regular flare-ups, a feature that was not noted in our patient. This case, therefore, represents a valuable example of an association between OCPs and IC in a premenopausal female.

In this patient, the diagnosis of IC was missed at several encounters in favor of UTI, nephrolithiasis, and vaginitis. The patient failed several rounds of antibiotics, antifungals, and analgesics over the course of a year. Originally, the National Institute of Diabetes and Digestive and Kidney Diseases (NIDDK) had created consensus criteria for the identification of IC for research purposes. These eventually became the de facto criteria for the diagnosis of IC and mandated the documentation of certain findings to support the diagnosis. These include either glomerulations or the classic Hunner’s ulcer on cystoscopy, associated with either bladder pain or urinary urgency. Because this definition left many patients undiagnosed and untreated, the NIDDK developed the Interstitial Cystitis Database Study, which removed the requirement of a baseline cystoscopy, making the diagnosis of IC one of exclusion based on a common pattern of symptoms. This patient presented with common symptoms consistent with a diagnosis of IC, along with a notable history of hormonal contraception use. Because of this, it is vital to examine existing research linking IC with OCP use in order to appropriately develop clinical suspicion for IC in females taking OCPs with unrelenting pelvic and bladder pain [13].

Very few studies discuss the possible impact of hormonal contraception on the pathophysiology of autoimmune disorders in women, even though many women use OCPs at some point throughout their lifetime. A literature review by Williams in 2017 examined the association between hormonal contraception and various autoimmune diseases, such as multiple sclerosis, ulcerative colitis, Crohn’s disease, systemic lupus erythematosus (SLE), and IC. The article presented two studies that linked the use of OCPs with the development of IC and demonstrated an increased risk of developing IC with OCP use [12].

The first of these studies was by Warren et al. in 2011 and reported the association between antecedent sexual/reproductive characteristics and IC. Results suggest a significant difference in usage of contraceptive hormones between IC cases and controls (88% vs. 82%; P = 0.019), with a mean duration of six years in the cases versus five years in the controls. This study also found that more IC patients reported non-contraceptive hormone use than controls (44% vs 25%; P = 0.001), with a mean duration of 2.4 years in the cases versus 1.6 years in the controls. This data demonstrates that there is a higher prevalence of IC in patients who use hormones, whether for contraceptive purposes or not, and supports the theory that estrogen contributes to the development of autoimmunity, specifically IC. In addition, this study shows that women with IC have a higher duration of hormone use compared with controls, which could inform clinical practice and duration of dosing of OCPs [4].

The second study was a case-control study conducted in Italy in 2008 by Gardella et al. to evaluate sexual dysfunction in women with IC compared to age-matched negative controls. The study reported a significant association between IC and current OCP use with an odds ratio (OR) of 6.9 [95% confidence interval (CI) = 2.1-22.1] and a significant association between IC and past OCP use with an OR of 4.6 (95% CI = 1.74-12.1) [14].

Another possible consideration regarding the effects of OCPs on IC is the effect of changes in testosterone levels. Vulvodynia and dyspareunia are common symptoms associated with IC, and though our patient did not report either of these symptoms, it may be important to consider them in relation to the pathophysiology of IC [15]. These symptoms are generally related to lowered testosterone levels, which is a known outcome of OCPs through the upregulation of sex hormone-binding globulin (SHBG), which binds testosterone. Considering our patient’s resolution of symptoms upon discontinuation of OCPs as well as the return of testosterone levels to normal after OCP discontinuation, it is possible that our patient’s symptoms were linked to a decrease in testosterone levels [16]. This could imply a connection between the course and symptomatology of IC and decreased testosterone levels.

## Conclusions

It is well-known that autoimmune disorders primarily affect women over men. It is also known that female hormones such as estrogens play a role in the development of autoimmune disorders through the upregulation of Th2 cells, which have been linked to a variety of autoimmune conditions as well as the lowering of testosterone levels. The studies previously mentioned, along with the current case, suggest that there is an association between IC and past or current hormonal contraception use. While these results are compelling, there remains a need in the scientific community to further investigate and establish this link. IC has been shown to be especially debilitating to patients, significantly and negatively affecting the quality of life. It is therefore in the best interest of these patients to build on the proposed association and work to better understand the possible negative effects of hormonal contraceptives on patients’ quality of life.
